# Temporal Statistics of Natural Image Sequences Generated by Movements with Insect Flight Characteristics

**DOI:** 10.1371/journal.pone.0110386

**Published:** 2014-10-23

**Authors:** Alexander Schwegmann, Jens Peter Lindemann, Martin Egelhaaf

**Affiliations:** Neurobiology & Cognitive Interaction Technology Center of Excellence (CITEC), Bielefeld University, Bielefeld, Germany; Lund University, Sweden

## Abstract

Many flying insects, such as flies, wasps and bees, pursue a saccadic flight and gaze strategy. This behavioral strategy is thought to separate the translational and rotational components of self-motion and, thereby, to reduce the computational efforts to extract information about the environment from the retinal image flow. Because of the distinguishing dynamic features of this active flight and gaze strategy of insects, the present study analyzes systematically the spatiotemporal statistics of image sequences generated during saccades and intersaccadic intervals in cluttered natural environments. We show that, in general, rotational movements with saccade-like dynamics elicit fluctuations and overall changes in brightness, contrast and spatial frequency of up to two orders of magnitude larger than translational movements at velocities that are characteristic of insects. Distinct changes in image parameters during translations are only caused by nearby objects. Image analysis based on larger patches in the visual field reveals smaller fluctuations in brightness and spatial frequency composition compared to small patches. The temporal structure and extent of these changes in image parameters define the temporal constraints imposed on signal processing performed by the insect visual system under behavioral conditions in natural environments.

## Introduction

While moving through a cluttered environment, animals need to gather information about their own movements as well as the spatial structure of their surroundings. The visual system is one important source of environmental information, but the eye reduces the spatial information to a two-dimensional representation of light intensities projected onto the lattice of photoreceptors. The visual system has to recalculate spatial information either by comparing images from the right and the left eye viewing the environment from different perspectives (stereopsis), or by evaluating motion parallax cues in the temporal image flow when moving through the scenery [1]. Fast-flying insects, such as flies and bees, rely on retinal image flow as a source of spatial information, because they need to respond to objects, such as obstacles or landing sites, at much larger distances than those that might be accessible by a potential stereoscopic mechanism, given the close distance of the two eyes, their low spatial resolution and the small overlap of their visual fields [2].

It is important to know the typical input received during behavior in natural environments, i.e. the image statistics of natural sceneries and the dynamics of changes caused by behavioral actions, to understand the mechanisms underlying vision. Visual systems of animals are thought to be adapted during the course of evolution to the specific spatiotemporal stimulus conditions encountered under the respective natural operating conditions. In particular, the mechanisms for extracting spatial information from the retinal image flow were concluded to have evolved in insects to optimally handle the information available in the natural input. This information is generated by an active flight and gaze strategy that is characterized by sequences of alternating saccadic turns and straight flight segments [2]–[13]. Retinal image motion conveys information about the depth-structure of the environment only if it results from translational self-motion, while rotational movements only cause depth-independent image changes [14]. Therefore, the saccadic flight and gaze strategy is thought to already separate the translational and rotational components of retinal image motion to a large extent at the behavioral level. This simplifies the extraction of spatial information from the retinal image flow.

Advances in technology have opened the possibility to record and analyze natural scenes, as has already been done in several previous studies [15]–[20]. Moreover, some earlier studies addressed not only the global statistics of static natural scenes (e.g. [16], [21]–[22]), but focused on the spatiotemporal image statistics during eye fixation movements in front of static scenes (e.g. [23]–[26]), the input of single photoreceptors to stimulus sequences recorded by a camera moving on an experimenter-defined trajectory [27], or to the reconstructed ego-perspective retinal image flow in specific natural or artificial behavioral situations ([17], [28]–[32]). However, none of these studies considered systematically the consequences of the distinguishing dynamics of the active flight and gaze strategy of insects.

Therefore, we analyze in the present study the temporal changes of image statistics evoked by movements mimicking the two most important prototypical movements of free-flying insects, with their characteristic dynamic features, forward-translations and rapid, i.e. saccadic, turns [2]–[13]. We recorded sequences of high-dynamic range panorama images on a trajectory reflecting the saccadic flight and gaze strategy of insects in an idealized form in a variety of cluttered natural environments. Image parameters, such as brightness, contrast and spatial frequency content, were analyzed across the different natural environments. The analysis was carried out for differently sized image patches and different viewing directions, because visual sceneries may vary tremendously, for instance, along their elevation (e.g. bright sky vs. structured ground with bushes and stones, etc.), and sensory analyzers may have differently sized receptive fields. We aimed at focusing on scenes to which an insect's visual system has most likely been adapting during evolution. Therefore, we analyzed environments devoid of any man-made structures, such as buildings or cars. In contrast to most previous studies, we characterized the temporal changes in image parameters elicited by translational and rotational self-motion mimicking the characteristic dynamics of the flight of insects, such as flies and bees, and, in this way, examined for the first time which changes the visual system of these animals were likely to encounter in natural environments. On this basis, it is possible to infer behaviorally relevant time scales of visual information processing.

## Methods

### Camera and spectral filter

**Figure 1:**
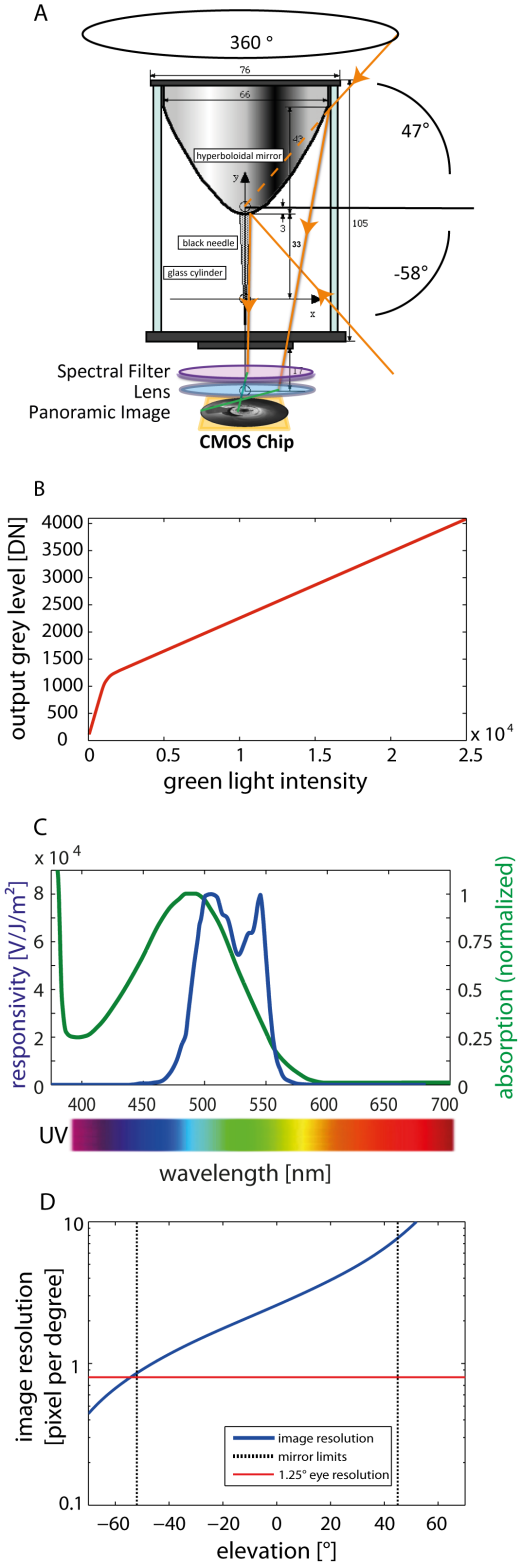
Panorama high-dynamic-range (HDR) imaging system. (A) Schematic side view of the panoramic mirror with optical path. The light rays (orange arrows) are reflected by the hyperboloidal panoramic mirror and passed through a spectral filter (see also inset) and a lens to form an image of the environment on the CMOS chip. The optical system is enclosed by a glass cylinder. A black vertical needle is fixed to the mirror to eliminate reflections caused by the glass cylinder. (B) Brightness transfer function showing the output grey level in DN (digital number) of the CMOS chip depending on the illumination intensity measured in the green spectrum at a wavelength of 480–560 nm (see Fig C, blue line) visualizing the LinLog feature and the intensity range of the camera. (C) Blue curve: Spectral bandwidth of the camera responsivity derived by the multiplication of the dichroic filter transmission characteristic and the raw CMOS chip responsivity. Green curve: normalized rhodopsin absorption rate of fly photoreceptors R1-R6 (data from [27]). (D) Image resolution (in pixels per degree) of the combined panoramic hyperboloidal mirror system and CMOS chip. The image resolution changes with elevation in the visual field as a consequence of the projection of the image by the mirror system onto the chip. The elevation range between the dashed vertical lines indicates the vertical extent of the field of view of the imaging system. The image resolution within this range is larger than 0.8 pixels per degree (corresponding to a spatial resolution of 1.25°, indicated by the red horizontal line) and, thus, better than the spatial resolution of the blowfly or honeybee eye.

We used a high dynamic range camera (PhotonFocus MV1-D1312-40-GB-12) which was mounted on a motor-driven linear feed and equipped with a panoramic hyperboloidal mirror (Accowle Vision HMN-X15) (Fig. 1A) to obtain image sequences.

The camera had approximately 1 Megapixel (effective usable size with our mirror: 928×928 pixels) and 12-bit A/D resolution. The sensor implemented an adjustable characteristic that was switching between two different gain values within one recording frame. This feature allows an encoding of a wide brightness range by approximating a logarithmic sensitivity characteristic via two linear segments of different slope (“LinLog-mode”) (Fig. 1B). Therefore, we could obtain a high dynamic range image with a single exposure instead of calculating an HDR image from multiple images with different apertures or exposure times.

We took great care to calibrate the CMOS chip of the camera properly. We recorded pairs of images from static sceneries comprising a large range of intensities to determine a calibration curve for the reconstruction of the true brightness from the image values. One such pair was taken with activated LinLog, the other with deactivated LinLog linearly encoding brightness. Pixels which saturated in the non-LinLog record were discarded. We were able to extrapolate the characteristic for high light intensities because the transition point between the low-brightness and high-brightness section of the characteristic was in the region measurable. Therefore, the slope of the linear high-intensity part could be determined from the measurements given and used for extrapolation. The resulting image values had a dynamic range of 1∶23,900 covering 3,955 intensity steps that resulted from 4,095 intensity steps of the 12-bit resolution, minus an offset of 140 DN (digital number; in image processing the output value of each pixel in a dataset) in the original LinLog image. The offset is caused by the camera calibration where no pixel should return 0 DN in darkness. Thus, we parameterized the CMOS chip in such a way that the pixels would return a value of about 140 DN in complete darkness.

We used a dichroic filter between the camera lens and the mirror to limit the camera's spectral sensitivity to wavelengths in the range of 480–560 nm. This filtering mimics the spectral sensitivity of photoreceptors R1-R6 that provide the input of the insect motion vision system (Fig. 1B,C) [32], [33]. As a consequence, however, we could not employ a unit like candela for characterizing brightness, but had to use the linearized digital return values of the pixels, which are, though being arbitrary units, proportional to light intensity in the green spectral range. In this paper, we refer to this unit as GLI (green light intensity).

### Panoramic mirror

We combined our camera with a hyperboloidal mirror, e.g. [35], to replicate the wide-field view of insects. The camera pointed upwards in the direction of the hyperboloidal mirror to cover the complete 360° panoramic view within a single image. Only the top view was blocked by the mirror itself, and the view straight to the bottom was blocked by the camera. A black needle was located in the center of the mirror to prevent double reflections caused by the glass cylinder. The final image covered the full 360° azimuth and an elevation between −58° below and 47° above the horizon.

The combination of HDR Camera and mirror has the advantage of capturing large parts of the panoramic visual field of an insect with one exposure. Thus, we were able to extract image patches for every viewing direction from only one image. Though image resolution drops for patches looking downwards, resolution is everywhere in the image above a spatial resolution of 1.25° (corresponding to 0.8 pixel/degree) and, thus, better than the resolution of the eyes of blowflies or bees (Fig. 1D; [35]–[38]). Alternative techniques such as generating a panorama by “stitching” images or computing HDR information from an image sequence generated by exposure bracketing require multiple cameras and/or exposures.

We used a slightly modified version of the omnicam-calibration toolbox by Davide Scaramuzza for Mathworks MATLAB 2010b [39]–[41] to calibrate the mirror geometry for the inverse projection of the image. It determines the geometry of the mirror from several images of a checkerboard pattern with known geometry. This procedure leads to (i) the geometric center of the images and (ii) the parameters to calculate the spherical coordinates of the viewing direction for any pixel in the image. As an extension of the toolbox, we created a method for finding an inverse function (*polyval*) to calculate the Cartesian source pixel coordinates for a given viewing direction needed for unwrapping via inverse projection of the circular image as provided by the hyperboloidal mirror system.

### Mechanical setup

The camera was mounted on a custom-made linear feed equipped with a stepper motor and placed at about half a meter above the ground. Camera height was chosen for pragmatic reasons, but was considered biologically plausible. Although no systematical research of outdoor flight height of blowflies is available, flies can easily be observed in this height range, but vary their flight height depending on the specific behavioral context. Camera height is likely to affect image statistics in a quantitative way, but we assume that the gradients analyzed along elevation will not change qualitatively. The results for lower regions of the visual field were expected to become more similar to those obtained at equatorial viewing directions the higher the camera was placed above the ground. The camera and the drive were remotely controlled. We recorded in sequences of 1-cm position steps on a linear path. On this basis, we computed a variety of rotational and translational image sequences. For technical reasons, subsequent images were taken at time intervals of 2 seconds, i.e. at a much lower frequency compared to a real motion of, for instance, 1 m/s. Thus, the translational image sequences obtained in this way correspond to those that would have been obtained during real motion only if the visual scenery had not changed (e.g. no brightness changes due to clouds occluding the sun or movements of leaves, etc.). We will argue in the Discussion section that this limitation does not affect the basic conclusions drawn in the paper. We selected recording sites in a wide range of different types of natural environments. The latter comprised diverse natural surroundings, such as cluttered forests, open fields or shrub land. We recorded 37 sequences with 100 images each. The GPS coordinates of the locations where the image sequences were taken will be provided together with the published image data.

### Projection and patches

**Figure 2:**
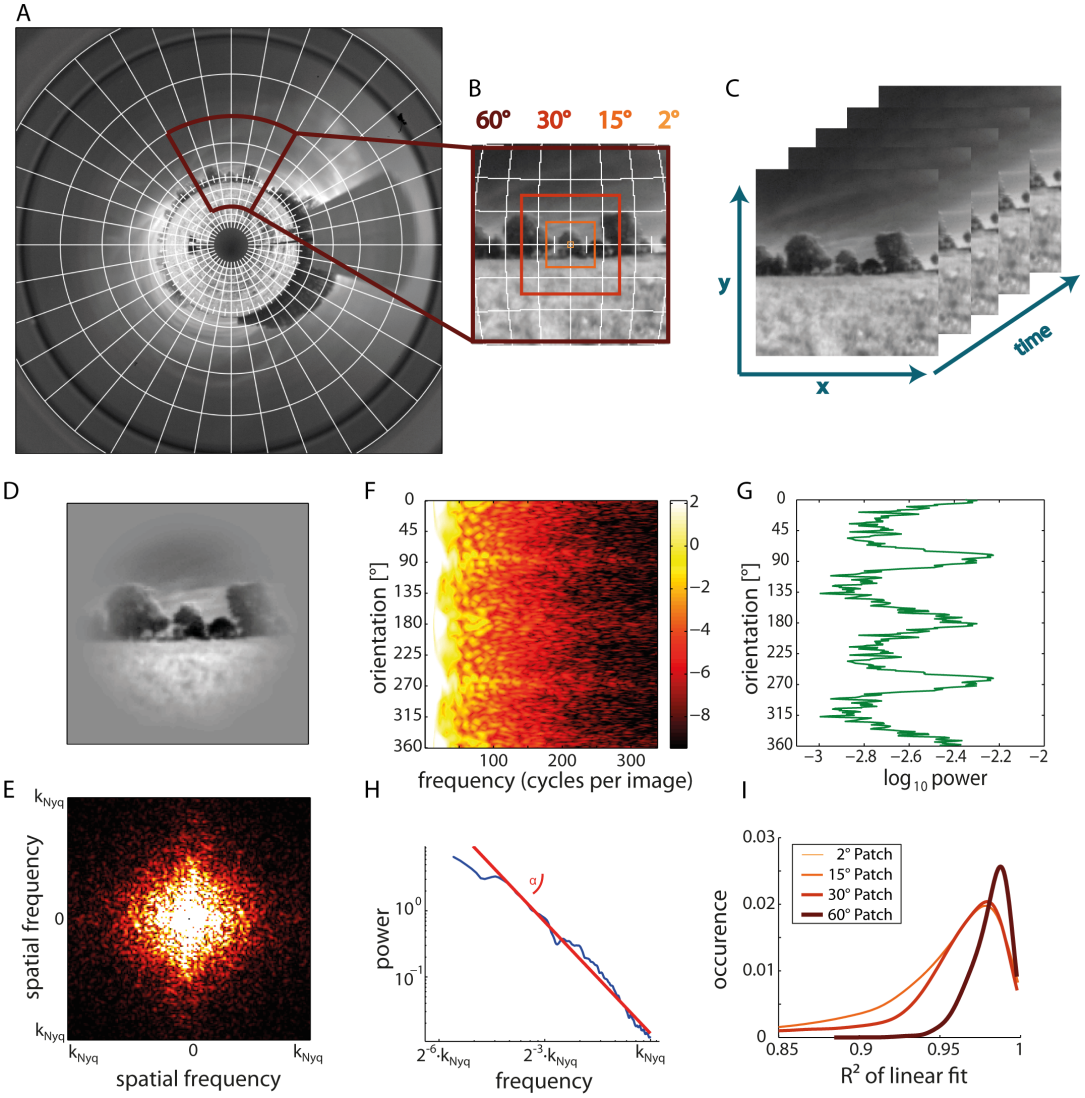
Analysis of image sequences. (A) Original ring image with overlaid grid. (B) Extracted image patch in the Lambert-azimuthal equal-area projection with the four different patch sizes of 2° (light thin box), 15°, 30°, and 60° (dark thick box). The color code is consistently used in the following figures. (C) Sequence of image patches with time as third dimension. (D) Tapered image patch. Before any image analyses were performed, tapering by a sinusoidal window had been applied to avoid boundary effects. (E) Two-dimensional fast Fourier transformation of a single frame; the color code gives the magnitude of the Fourier components with brighter colors indicating larger magnitudes. Spatial frequency is increasing with distance from the figure center. (F) Color-coded power spectra obtained from (E) showing the power for the different orientations and frequencies in the image. (G) Total power per orientation obtained by taking the mean for the rows in (F). (H) Total power per frequency obtained by taking the mean across columns in (F). Linear fit visualizes the 1/f^α^ characteristic common for natural scenes. (I) Histograms of the goodness of the linear fit to the frequency-dependent power spectra as plotted in (H) for all image patches of our dataset showing the quality of the 1/f^α^ frequency dependence in our dataset of natural images.

Biological mechanisms of visual information processing collect the visual input within receptive fields of a wide range of diameters and at different locations in the visual field. Therefore, we scrutinized the statistics of the natural image sequences within image patches of variable size and location in the visual field. Patch diameters amounted to 2°, resembling the acceptance angle of an insect's ommatidium, as well as to 15°, 30° and 60° (Fig. 2A–C). The centers of these patches were located at elevations of −30°, 0° and 30° relative to the equator of the visual sphere. We placed the patches in 30° steps from −90° azimuth (looking to the left) to 90° (looking to the right). Additionally, we placed patches at −45° and 45° azimuth. The patches with a 60° aperture could only be placed at the equator because they would have overlapped with the regions which were out of sight of the camera at elevations of −30° and 30°. In total, we employed 90 patches for each panoramic image.

The patches were determined with the Lambert azimuthal equal-area projection. This projection provides equal areas with the least central distortions and has the following advantages: Firstly, an equal area does not affect the size of objects at different elevations. Thus, objects in the upper or lower regions of the image do not have a different impact on image statistics. Secondly, the central longitude and central latitude of the projection can be freely selected. Thirdly, the Lambert azimuthal projection, though not being isogonic and causing heavy distortions at the outer projection regions, provides minimal distortions for image parts taken at the projection center. Shape is only minimally distorted (less than 2% within 15° from the focal point) and, therefore, the image patches used in this study can also be considered as nearly angle-preserving. Though the type of projection may influence image statistics, e.g. [42], this projection was considered as the one best suited for our purposes and least influencing image statistics for small image patches. However, in more eccentric regions, angular distortions would become more significant for this projection: small shapes are compressed radially from the center and elongated perpendicularly. We ignored these distortions because we only analyzed image patches in the undistorted range.

We used ordered grid super-sampling antialiasing [43] with a 9×9 grid to minimize aliasing artifacts resulting from discretization to integer image coordinates. This procedure neither blurs nor sharpens the image. Instead it prevents the accruement of jaggies, an aliasing artifact caused by sampling one raster image onto another grid with different geometry.

### Statistical analysis

We analyzed the time course of first order statistics of the mean brightness and of the root mean square contrast in differently sized image patches during rotational and translational movements (see Appendix S1 for details). Moreover, we examined the spatial frequency statistics within the image patches and, thus, calculated two-dimensional power spectra (Fig. 2D–F), after having tapered the image patches with a sinusoidal window to eliminate boundary effects. Power spectra were calculated by taking the squared magnitudes of fast Fourier transformations (FFTs). Taking the mean over all frequencies for each viewing direction, we obtained the orientation-dependent power spectra (Fig. 2G) providing some information about the orientations of contours that were most prominent in the image. By taking the mean over all orientations for each frequency, we calculated the frequency-dependent power spectra (Fig. 2H). More details are given in Appendix S1.

The spectral power of natural images was found in previous studies to approximate, on average, the function 1/f^α^, with f corresponding to the spatial frequency and α being an exponent [44]. This decrease of power with increasing spatial frequency was described to comply with α varying widely around 2 [16], [45]–[46].

We calculated the different image parameters for each image patch separately. However, no reliable power spectra and, thus, no α could be determined for the 2° patch due to its small size. The overall quality of the linear fits decreases with decreasing patch size, although the peak of the corresponding R^2^ distributions is still above 0.95 for path sizes as small as 15° (Fig.2I).

## Results

Image parameters such as brightness, contrast and the spatial frequency spectrum may vary within a given patch of the visual field over time during flight in natural environments. Due to the fact that the saccadic flight style of insects separates translational from rotational movements, the parameter variations can be expected to differ substantially for these two movement prototypes. In order to understand how apparent such differences are in natural environments and how much they may vary between different types of environments (e.g. forest vs. open terrain), we analyzed the amplitude and speed of fluctuations in the different image parameters during translational and rotational movements with a dynamics mimicking that of the free-flight of insects, such as blowflies and bees. We placed particular emphasis on the fluctuations caused by translational movements because the corresponding optic flow contains depth information. Although the fluctuations during translations appear to be more informative for the animal, the visual consequences of saccade-like rotations may also be relevant, because the image parameter in a given patch of the visual field may differ a lot between the start and end of a turn, depending on the type of environment. The sensitivity range of the visual system and the information-processing mechanisms of an animal need to be adjusted to consider these characteristic changes in image parameters.

**Figure 3:**
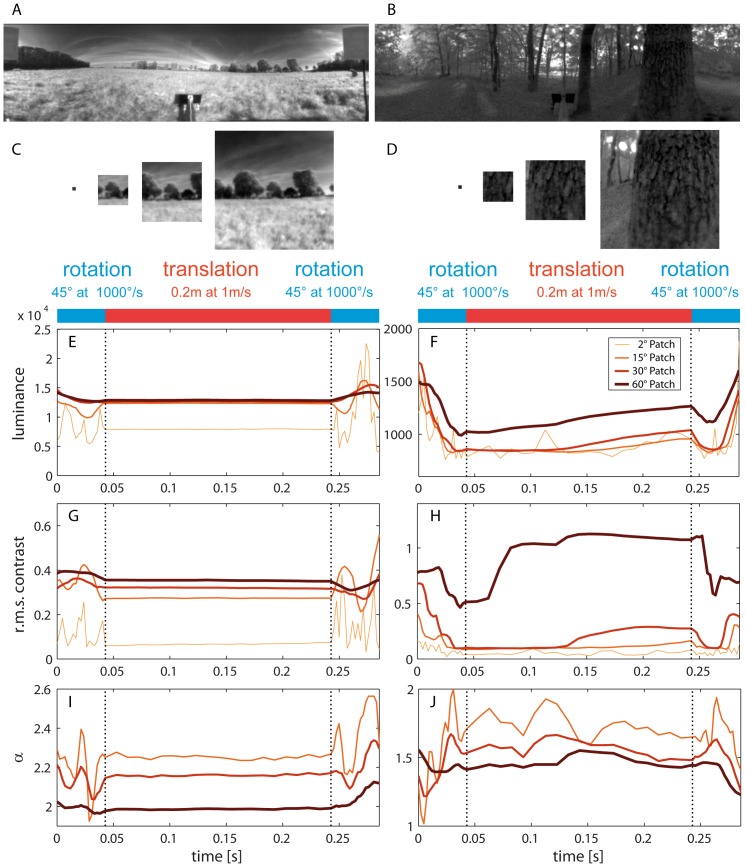
Sample time courses of image parameters during naturalistic rotational and translational movements in two environments. Movement consists of a 45° rightward rotation at 1000°/s followed by a 20 cm translation at 1 m/s, while looking 90° to the right, followed by another 45° rightward rotation. (A, B) Single full-panoramic sceneries of one frame of the two image sequences. (C, D) The differently sized image patches for a single frame. (E, F) Time course of mean brightness for all four patch sizes. (G, H) The root mean square contrast versus time. (I, J) Time course of the α-exponent of regression line fitted to power spectra (no time course is plotted for the 2° patch because a reliable Fourier transform estimation was not possible with such a small patch size). Dotted lines mark the beginning and the end of the translational phase. Color codes for patch size (cf. Fig 2B).

Rotational movements as fast as saccades of insects can be expected to cause at least, on average, faster image displacements than intersaccadic translational movements. Nonetheless, fast image displacements may also be found during translation when passing, for instance, a nearby object, while rotations in an open field may only lead to small changes. A first impression of the time courses of image parameters, while moving through two different sample environments, an open field scenario and a forest scenario, is provided by Figure 3. The fluctuations of stimulus parameters result from an initial 45° rightward saccade-like rotation on the spot followed by a 20 cm translation, while looking 90° to the right at 0° elevation, and then another 45° rightward rotation on the spot.

The mean values of image parameters (especially brightness and the contribution of high spatial frequency as expressed by the α-exponent) differ considerably between the different types of environment. The fluctuations of the image parameters analyzed around these mean values are considerably stronger and faster during saccade-like rotations than during translations (Fig 3E–J). This is particularly prominent in the open-field scenario. During translations, fluctuations are only prominent for the forest scenario containing objects, e.g. trees close to the camera. Fluctuations appear to become larger, the smaller the aperture of the patch analyzed. These examples already indicate that it is not easily possible to predict the environment in which the motion was performed from the fluctuations of the image parameters. However, large and fast fluctuations strongly hint at rotational movements (see below). Since the two sample scenes analyzed in Fig. 3 are only the two ends of a kind of continuum (open field vs. dense forest), we did not try to classify sceneries.

### Distribution of image parameters in natural sceneries

**Figure 4:**
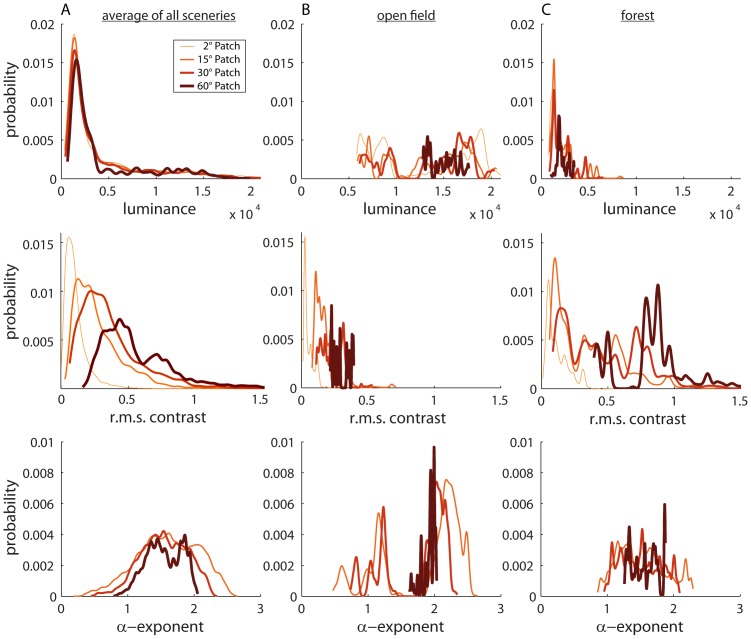
Probability distributions of image parameters for the differently sized image patches. Analysis of the entire dataset (A) and of the two sample sceneries (B) “open field” and (C) “forest” shown in Fig. 3. Upper diagrams: Probability distribution of brightness; middle diagrams: rms contrast; bottom diagrams: α-exponent. In the plots we show the running average of the very spiky original histograms calculated with a Gaussian average window using a window size of 1/25 of the bins (500 bins).

The brightness in natural environments is, on average, approximately log-normally distributed (Fig. 4A, upper traces) or, more precisely, sub-Gaussian [6]. Changing the patch size does not change the overall brightness distributions significantly (Mann-Whitney-U Test, 60° versus 30°: p = 0.0884; 30° versus 15°: p = 0.3498; 15° versus 2°: p = 0.2660). However, we found substantial differences between brightness distributions when comparing different surroundings. It is significantly brighter in the open field environment (Fig. 4B, upper plot), for example, than in a forest (Fig 4C, upper plot) (p<0.0001).

The contrast distributions depend on the patch size, which is in line with previous work [24], [26]. With an increasing patch size, a wider range of brightness levels is more likely to be covered by the patch; therefore, the rms contrast tends to increase with the patch size (p<0.0001) (Fig. 4A, central plot). The mean contrast distributions are not significantly different from a lognormal distribution (p>0.05; one-sample Kolmogorov-Smirnoff test; detailed parameters shown in Table 1). The contrast distributions also depend on the environment. We found much higher contrasts in the forest than in open fields, most likely due to objects (trees, etc.) in front of a bright sky (Fig. 4B, middle plot; Fig. 4C, middle plot).

**Table 1:** Parameters of lognormal contrast distribution.

Patch Size	μ	σ^2^	n	d_max_	d_α = 0.05_	d_α = 0.95_	p
2°	−2.488	0.762	973	0.0408	0.0435	0.0196	0.0566
15°	−1.464	0.612	973	0.0245	0.0435	0.0196	0.5999
30°	−1.194	0.604	973	0.0273	0.0435	0.0196	0.4353
60°	−0.666	0.425	325	0.0392	0.0753	0.0392	0.6857

Parameters of the lognormal distributions of the r.m.s. contrasts of our database. μ is the expected value, σ^2^ the variance of the distribution; both parameters were obtained by a lognormal fit to the distribution. n is the number of elements. The other values give the significance obtained with the one-sample Kolmogorov-Smirnoff test, with d_max_ being the maximal deviation of the empirical function from the data and the estimated lognormal distribution and d_α = 0.05_ the critical value for the null hypothesis: if d_max_ is larger than this value, the hypothesis that the distribution is lognormal can be rejected. d_α = 0.95_ is the critical value for the alternative hypothesis: if d_max_ is smaller than this value, the alternative hypothesis that the distribution is not lognormal can be rejected. The corresponding p values are given in the last column.

The spatial frequency spectrum also depends on the patch size. The exponent α, characterizing the slope of the decline in spectral power with increasing spatial frequency, was found, on average, to be α = 1.57±0.39 for our dataset. However, there are systematic differences between different types of scenes, as has already been observed in other studies (e.g. [47]–[48]). We found α = 1.8 for open field environments and α = 1.4 for forest-like environments. Hence, high spatial frequencies are more prominent in cluttered environments than in open areas with an unimpeded view of the sky. Changing the patch size does not change the mean α, which is in line with scale invariance, i.e. local quantities in natural scenes, such as frequency composition, do not change by scaling [46]. However, bigger patches reduce the standard deviation of α (Fig. 4A, bottom plot). Therefore, α values are more variable and larger with smaller fields of view, though the mean over all scenes stays the same.

### Orientation bias of environmental structures

**Figure 5:**
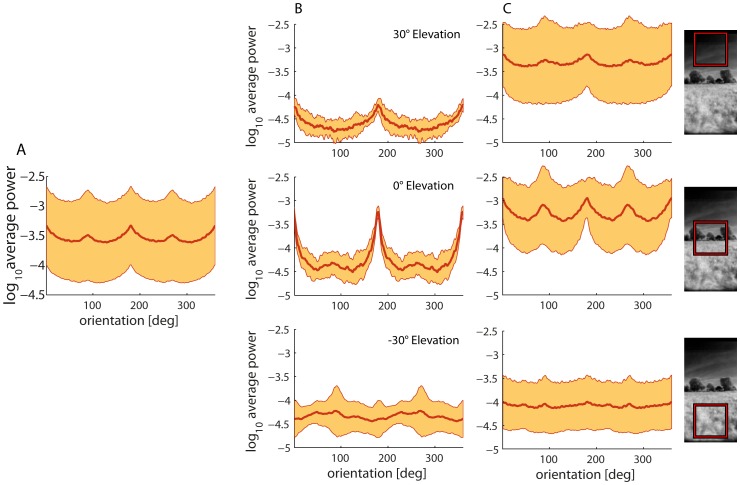
Mean power versus orientation obtained for 30° patches. (A) Mean power (thick line) and standard deviation (marked area) across all images in the dataset. Note the predominance of horizontal and vertical orientations in natural images. (B) The power per orientation for the open field example (cf. Fig. 3A) at different elevations as indicated in the subfigures. (C) Power per orientation for the forest example (cf. Fig. 3B) at different elevations. Note the considerable differences between elevations and between different natural environments.

The orientations of contours in natural environments are not uniformly distributed. In accordance with previous research [16], [49]–[50], most power is found for vertical and horizontal orientations when pooling our whole dataset for a patch size of 30° (Fig. 5A). We analyzed image patches of the two sample scenes separately for different elevations (−30°, 0° and 30°) (Fig 5B and 5C) to assess whether the orientation of spectral power depends, on the one hand, on the environment or, on the other hand, on the elevation in the visual field, as has already been carried out in several previous studies [48], [51]–[52]. The power per orientation differs substantially between the different elevations and for the two sceneries. The horizon, for example, contributes by far the most prominently oriented power in the open field (Fig. 5B, middle plot). However, in the forest scenery, the horizon contributes approximately the same power as vertical contours, but all other orientations are also much more dominant than in the open field (Fig. 5C, middle plot).

**Figure 6:**
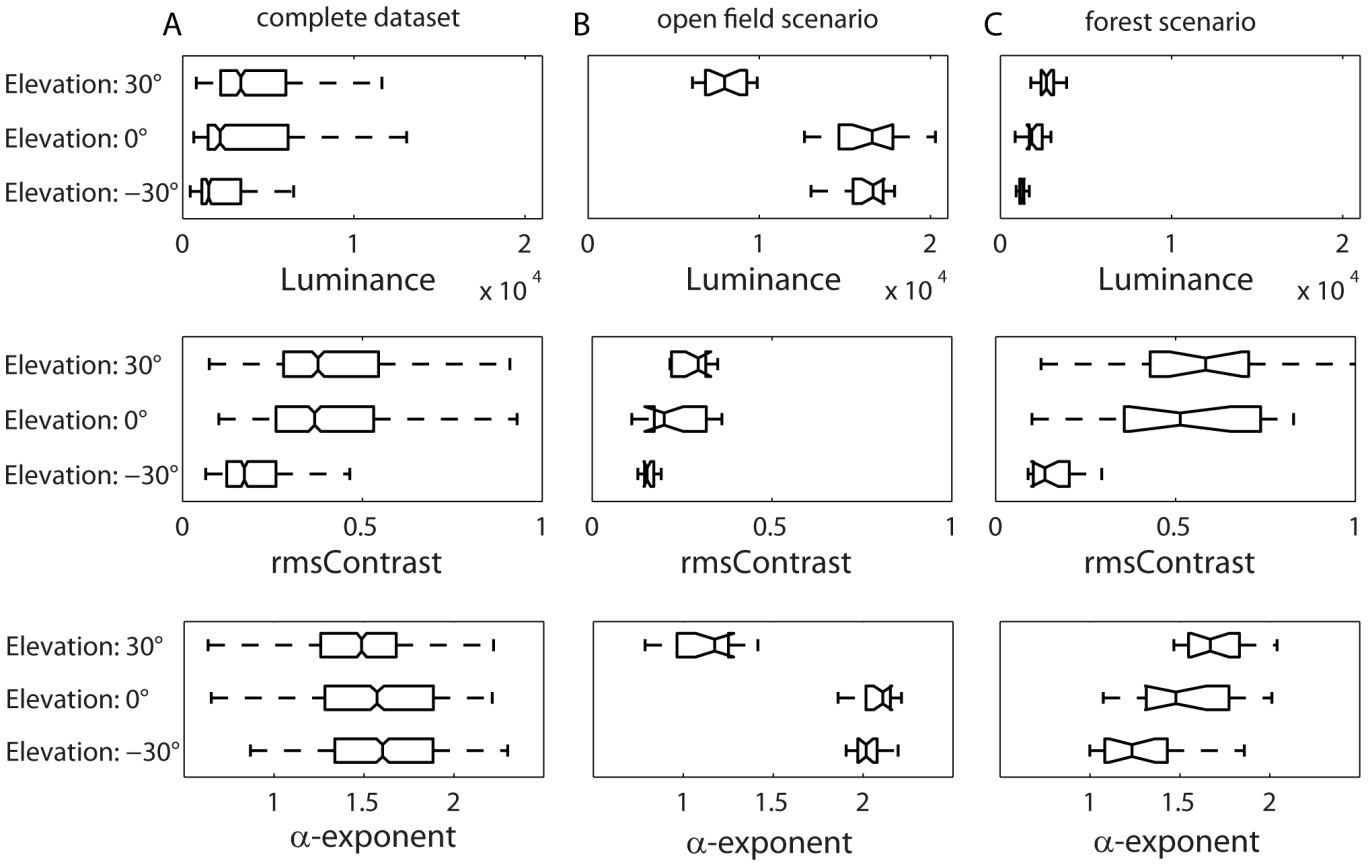
Box-and-whisker plots of the distribution of image parameters at different elevations in the visual field. (A) For the entire dataset, (B) for the “open field” example and (C) for the “forest” example. (Upper diagrams) brightness, (middle diagrams) rms contrast, (bottom diagrams) α-exponent. Boxes indicate the 25th (left boundary), 50th (median, center line of the box) and 75th (right boundary) percentiles. Whiskers show the minimum and maximum disregarding the outliers. Outliers are not shown in the plots.

No orientation predominates in the power spectrum averaged across all natural environments analyzed in the ventral visual field. Horizontal orientations are apparent in the dorsal visual field – though not as many as when looking at the horizon. The power of every orientation in the upper part of the forest scene is significantly stronger than in the open field. This is probably caused by the treetops adding a lot of edges in every direction. Therefore, the absolute power across orientations is higher compared to the sky of open fields, which correlates with the higher contrast in this part of the visual field of the forest scene (Fig. 6B, middle plot and 6C, middle plot).

Though it has previously been shown that projections may influence the image statistics over the hemisphere, and may cause anisotropies [42], we avoided such anisotropies by choosing the Lambert azimuthal equal-area projection. We ensured that the image patch always covered the same area with less than 2% distortion by continuously calculating a new projection for each patch centered on the respective viewing direction and by analyzing the image parameters in the least distorted region, i.e. in the center of the projection.

### Elevation dependence of image parameters

Since natural scenes often appear to be characterized by inherent asymmetries along their elevation (e.g. sky in the upper part of the visual and ground structures in the lower part), we investigated whether the different image parameters, i.e. brightness, contrast and α-exponent, depended on the elevation in the visual field (Fig. 6). This analysis was carried out for 30° patches using a Mann-Whitney U Test with Bonferroni correction for statistical analysis.

By taking the entire database into account (Fig. 6A), larger brightness values predominate in upper image regions. Accordingly, the ground is, on average, darker than more elevated regions (p<0.0001 for all comparisons). A similar gradient is visible for the contrast. At an elevation of −30° (lower image parts), images show significantly lower contrasts than at the other elevations analyzed (p = 0.1966 for 30° versus 0°; p<0.0001 for −30° versus 0°; p<0.0001 for 30° versus −30°).

Alpha-exponents in the upper part of the visual field are significantly smaller than in the equatorial parts, while they do not differ significantly in lower image regions (p<0.0001 for 30° versus 0°; p = 0.0586 for −30° versus 0°; p<0.0001 for 30° versus −30°). Since the distributions of α-exponents in the different eye regions overlap tremendously, we have not considered this difference to be functionally relevant.

Whereas the ground contrast is significantly lower for all environments than the contrast in other image regions, individual sceneries may differ greatly from the average in this regard. Gradients can even be reversed in some environments (compare Fig. 6B and 6C). In the spectral range analyzed (mainly “green”) that mimics that of the insect motion vision pathway, for instance, the sky in open field sceneries may be darker than the other parts of the image (Fig. 6B, upper plot). Moreover, individual scenes differ significantly, although, on average, we found no elevation-dependent gradient for the α-exponent. In the open field example (Fig. 6B, bottom plot), for instance, α-exponents in the equatorial and lower regions of the image are higher than in the sky region, indicating more power in the high frequency range in the sky than on the ground. This finding is counterintuitive at first glance, however, the many thin white cirrus clouds in the respective scenery (Fig. 3C) have contributed considerably to the elevated power in the high spatial frequency range (Fig. 6B bottom plot). The elevation dependence of α in the forest environment example is reversed (Fig. 6C bottom plot).

### Fluctuations of image parameters during simulated movements

This study focuses on how rotational and translational self-motion in natural environments with a time course reminiscent of that of flies and bees shape the time course of brightness, contrast and spatial frequency content in different parts of the visual field and differently sized image patches. Since we were mainly interested in the statistics of image sequences resulting from motion in the environment, the spectral range of the images was adjusted to that of the insect's motion vision system. The amplitude of the time-dependent fluctuations was quantified for all parameters by determining the standard deviation across time, while either the camera translated by 1 meter or the panoramic image was rotated by 360°. We computed the mean of the absolute value of the first time derivative of the parameter values to determine the speed of fluctuations of the parameters.

**Figure 7:**
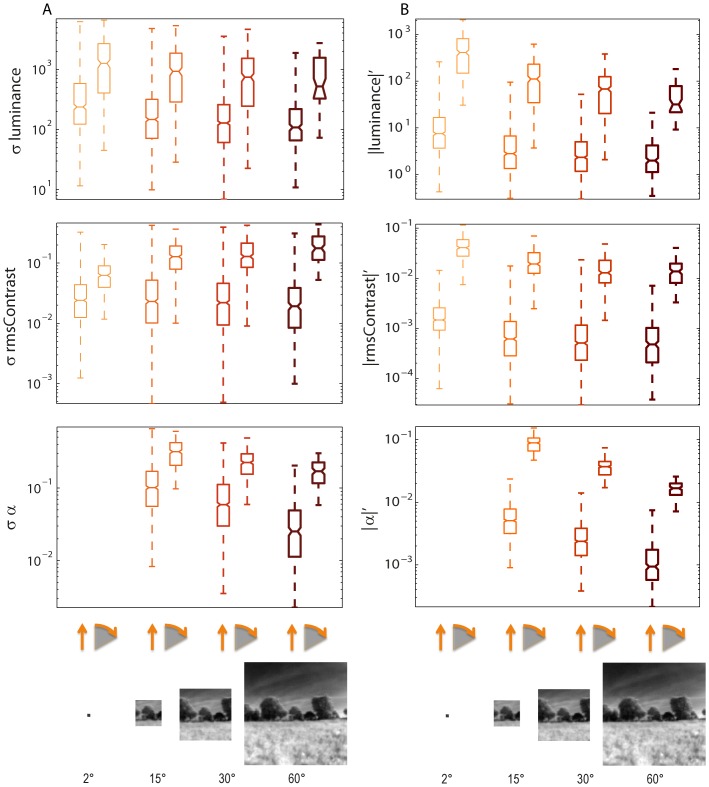
Amplitude and speed of time-dependent fluctuations of image parameters. Data are shown for rotational or translational movement and for different image patch sizes. (A) Fluctuation amplitudes during 360° rotations at 1000°/s and translations for 1 m at 1 m/s (as indicated below diagrams) Fluctuation amplitudes are given as standard deviation across time. (B) Speed of fluctuations for the same flight dynamics as in (A) measured by taking the mean absolute first derivative of the time courses of image parameters. Upper diagrams: brightness, middle diagrams: rms contrast, bottom diagrams: α-exponent. Each pair of boxplots shows fluctuations caused by rotation in comparison to translation (see insets below). Patch sizes are shown in different colors and line thickness as before.

On average (Fig. 7), but also for individual sceneries (see Fig.3), the amplitude and speed of all image parameters fluctuate much more during rotational saccade-like movements than during translational ones. The fluctuation amplitudes differ by about one order of magnitude (Fig. 7A). The difference in fluctuation speed is even larger (Fig. 7B). The amplitude and speed of fluctuations decrease with increasing patch sizes. The only exception is the amplitude of contrast fluctuations which tend to increase with increasing patch sizes during saccade-like rotations, but tend to change only little during translations (Fig. 7A, middle plot). Mean brightness and the α-exponent larger patches, meaning a larger integration area, tend to reduce the amplitude of fluctuations. For contrast fluctuations, however, large patches are likely to increase fluctuation amplitudes, because they are more likely to slide over differently illuminated image regions during rotations than small ones. Contrast fluctuations are smoother during translations than during saccade-like rotations. While small image patches are accompanied by rapid, but small contrast fluctuations, these fluctuations become smoother and larger with increasing patch sizes. The smoothing effect of large patch sizes is most prominent for the α-exponent (Fig. 7B, bottom plot).

### Overall changes of image parameters during simulated movements

**Figure 8:**
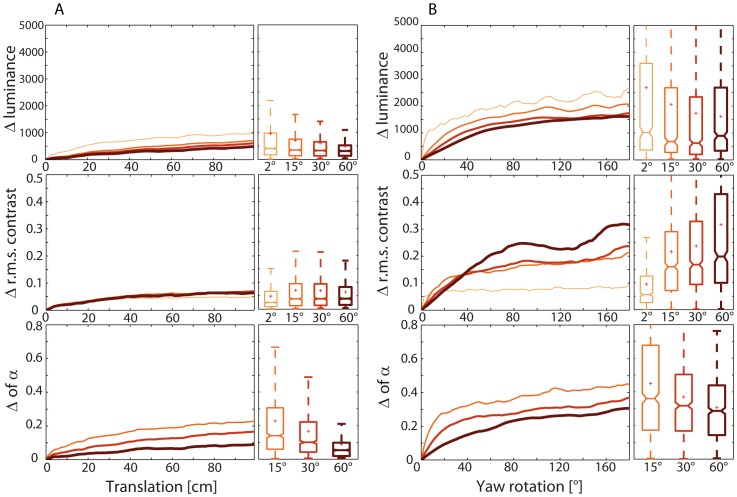
Mean absolute overall changes of the three image parameters for different patch sizes. Data are shown as a function of (A) translation distance and (B) of overall amplitude of rotation. Upper diagrams: brightness, middle diagrams: rms contrast, and bottom diagrams: α-exponent. Parameter value of the first image always set to zero; plots share a common scale for better comparison. Distributions of overall parameter changes across the different image sequences shown in the boxplots after (A) a 1-m translation and (B) a 180° rotation. Patch sizes are encoded by color and line thickness as before.

Insect flight behavior causes changes in brightness, contrast and spatial frequencies on the retina. These changes differ between saccades and intersaccadic translations. We determined the absolute difference between the initial values of brightness, contrast and α-exponent at the onset of either the rotation or translation and the corresponding values at subsequent times during the respective movement to quantify the overall changes in the image parameters resulting from a simulated saccadic rotation of a given size or a simulated translational displacement over a given distance. We tested translations of 1 m in comparison to leftward as well as rightward rotations of 180°. The consequences of rotations were analyzed for the first, the middle and the last image of the 1-m image sequence. We did not rotate each image of a sequence, because the panoramic images at neighboring locations are very similar. We then determined the mean as well as the distribution of overall motion-induced changes of the different image parameters as a function of translation distance (Fig. 8A) and rotation amplitude (Fig. 8B).

Rotations in an amplitude range typical for saccadic turns of insects usually cause larger overall changes of image parameters than translations in the range plausible for intersaccadic movements do. Larger patch sizes tend to reduce the overall changes in brightness and the α-exponent. Overall contrast changes during translations appear to be unaffected by the patch size (Fig 8A, middle plot), while they even increase during rotations with increasing patch sizes (Fig 8B, middle plot).

**Figure 9:**
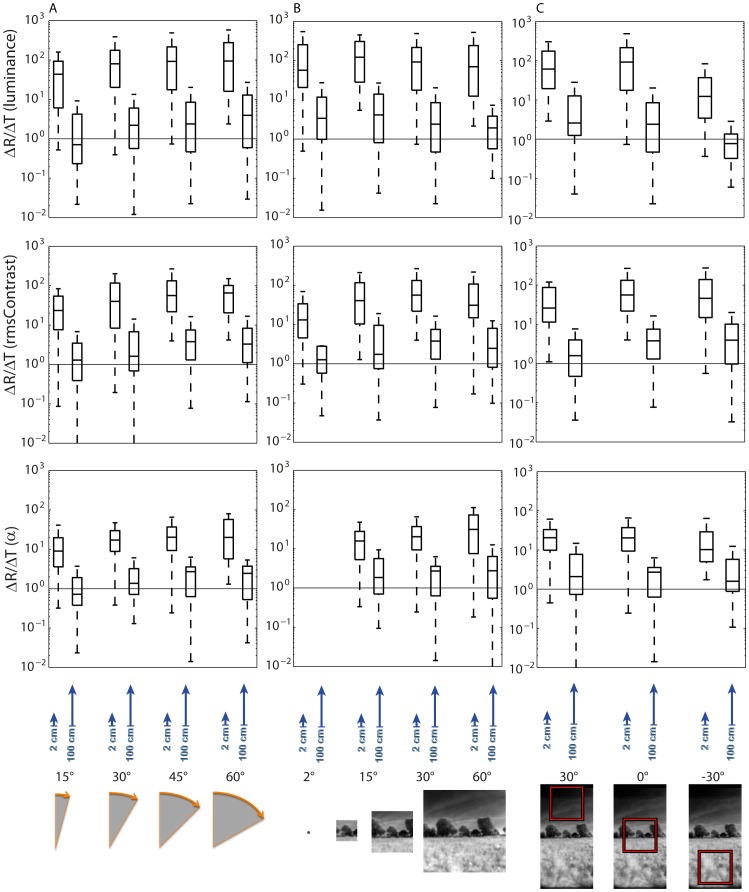
Comparison of ratio between the changes of the image parameters analyzed. Changes in brightness (upper row), rms contrast (middle row) and α-exponent (bottom row) are shown as caused by rotations (ΔR) and by translations (ΔT) over either 2 cm or 100 cm (indicated by blue arrows underneath diagrams). Rotations and translations start at the same image of a sequence. Horizontal lines in all diagrams illustrate the ratio of 1 where the changes induced by rotation and translation are the same. (A) Rotation amplitude was varied in 15° steps (see inset below diagrams). Patch size (30°), elevation (0°) and azimuth (+90°) of patch center were constant. (B) Patch size was varied (2°, 15°, 30°, and 60°, as indicated below diagrams). Rotation amplitude (45°), elevation (0°) and azimuth (+90°) of patch center were constant. (C) Elevation of patch was varied as indicated below diagrams (+30°, 0°, −30°). Patch size (30°), rotation amplitude (45°) and azimuth (+90°) of patch center were constant.

We calculated the total parameter changes caused for two extremes of translation distances (2 cm and 100 cm) and a range of rotations (15°, 30°, 45°, and 60°) at 0° elevation and a patch size of 30° to further assess the relative difference between overall changes in image parameters caused by rotational and translational movements, respectively. We determined the ratio between the absolute changes caused by the rotation specified (ΔR) and by the translation associated (ΔT) for all eight possible combinations (Fig. 9A). Apart from the most extreme case of comparing a 100-cm translation to the smallest rotation of 15°, the median ratio is larger than 1, indicating a bigger change induced by the rotation than by the translation.

In a second analysis, we fixed the rotation amplitude to 45° and altered the patch size (2°, 15°, 30°, and 60°: Fig. 9B). The patch size does not have any obvious influence on the ΔR/ΔT ratio, which is in line with the results from the fluctuation analysis shown in Figure 7.

In a third approach, we fixed the rotation amplitude to 45° and the patch size to 30°, and altered the elevation of the patch analyzed in the visual field in steps of 30° from −30° to 30°. The ΔR/ΔT ratios are smaller for the large translations than for small ones for all image parameters and all elevations analyzed. Whereas ΔR/ΔT values are fairly independent of elevation for the spatial frequency content and contrast, they appear to be slightly smaller for the overall brightness changes at the lowest elevation.

In summary, changes in image parameters induced by saccade-like rotations are, in most cases, larger than those induced by translations. Only when the consequences of small rotations are compared to large translations, may the parameter changes be similar.

## Discussion

Visual systems have to cope with the peculiarities of the image statistics encountered in behavioral situations, i.e. the brightness, contrast and textural features of the retinal input, as well as their characteristic temporal changes during behavior in natural environments. Therefore, they are thought to be adapted to these natural operating conditions by evolution. Although natural image statistics have been widely analyzed (e.g. [15]–[16], [18], [21], [53]), only a few studies took into account that under behavioral conditions, retinal image statistics are dynamic and that the dynamic changes are shaped, to a large extent, as a consequence of the action-perception cycle by the animal's behavioral actions [17], [23]–[32]. Therefore, we addressed this issue for the first time systematically with respect to the specific dynamics of the retinal image flow as is characteristic of the saccadic flight and gaze strategy of insects, such as flies, wasps and bees [3]–[12]. Since we were mainly interested in the statistics of moving image sequences that form the basis of spatial vision in flying insects by providing motion parallax cues [2], the visual input was characterized in the spectral range of the motion vision system of insects ([33]–[34], [54]–[56], see also [57]). Consequently, brightness and contrast gradients may differ a lot from what humans experience with their photopic system: for instance, a blue sky on a sunny day that appears very bright to humans was found to be darker than a green meadow when analyzed in the spectral range of the insect motion vision system.

Most importantly, we could show that saccade-like rotational movements usually elicited much larger changes in brightness, contrast and spatial frequencies than translational movements. Distinct changes in image parameters are suggested to be elicited during translations, mainly by nearby objects. In the following, we will (i) compare the characteristics of image parameters in our dataset with previously published results, (ii) identify characteristic changes in image parameters resulting from naturalistic self-motion, and (iii) address the consequences of the characteristic image dynamics during saccades and intersaccadic intervals of insect flight for the processing of retinal image flow and for neuronal adaptation.

### Characteristics of natural sceneries

The brightness, contrast and spatial frequency composition differ greatly between different natural environments, but also vary within a given scenery. The distribution of image parameters also depends on the patch size within which the sceneries are analyzed. Different patch sizes may correspond to differently sized receptive fields of neurons involved in visual information processing. On average, we did not find any dependence of the brightness distribution on patch size. Contrast reveals, on average, a lognormal distribution across all patch sizes, with larger patches resulting in larger contrast values. This is also true for the distribution of the parameter describing the decline in spectral power with increasing spatial frequencies (α-exponent): it depends on the patch size, with smaller patches resulting in more variations across the images.

We found a contrast gradient for most sceneries along the elevation of the visual field, with higher contrasts in its upper parts (Fig. 6), for our database of natural image sequences. However, Betsch et al. [17] found the contrast decreased with increasing elevation. This difference between studies can be explained by the spectral bandwidth of our camera system that we chose to mimic the spectral sensitivity of the flies' motion vision system, while Betsch et al. [17] analyzed video signals as seen by cats and used color images that were subsequently transformed into grey level images by differentially weighing the color channels (0.3 red, 0.6 green, 0.1 blue). The blue sky appears relatively dark for the motion vision system of insects, because – depending on the time of the day – the range of wavelengths where the motion vision system is most sensitive does not predominate in sky light (Fig. 6B). Since clouds scatter light in the whole visible bandwidth – they appear white – they may lead to high contrast borders in the sky region of open field sceneries and, thus, stimulate the fly's motion vision system. The fly's motion vision system is also sensitive to ultraviolet light [33]–[34]. However, the blue sky does not contain large amounts of ultraviolet light in the sensitivity range of fly photoreceptors because of the intense scattering and absorption of the UV light by the earth atmosphere [58]. UV-A light from direct sunlight scattered by the atmosphere may be detected by photoreceptors viewing the sky. The resulting UV-to-green contrast defining the horizon in natural environments was proposed to be useful in the context of visual navigation [59]. However, the relative proportions of green and UV components in the ambient light require UV and green sensors to have very different states of adaptation, because UV intensity in the sky is only a fraction of the green reflected by the ground. Therefore, we assume that photoreceptors feeding the fly motion vision system will not respond strongly to the UV scattered by the sky if adapted to the much higher intensities in the green wavelength range (see also [60]). Since green leaves and green grass appear as bright to the fly's motion vision system and even brighter than the blue sky, the brightness gradient in open field scenarios (Fig. 6B) may be inverted relatively to, for example, a forest scenery (Fig. 6C), where the ground is not covered with grass and, therefore, appears much darker than the equatorial and upper regions of the scene containing bright leaves and the sky.

The orientation bias of contours in natural sceneries, though found to a different extent in different environments and different parts of the visual field, has its correspondence in a similar bias for horizontal or vertical edges in the sensitivity of visual interneurons of a variety of animals, e.g. [61]–[64]. This feature can be regarded as an evolutionary adaptation of orientation sensitivity of visual systems to the predominant orientations of contours in natural environments.

The distribution of spatial frequencies in our database matches earlier findings. Van der Schaaf and van Hateren [16] found an average α-exponent of 1.88±0.43; Tolhurst et al. [53] found a mean value of α equivalent to 1.2±0.13 for their databases. In our database, the mean α-exponent of 1.57±0.39 is between these ranges. Although we did not find any elevation dependence of α on average, strong elevation-dependent differences and variations are observed in some environmental settings.

### Dynamical changes in image parameters resulting from naturalistic rotational and translational movements

The characteristics of static images only reflect to some extent the visual input of a behaving animal under natural environmental conditions. Therefore, we analyzed sequences of natural panorama images mimicking what a free-flying insect might perceive during translational and rotational self-movements as is characteristic of their saccadic flight and gaze strategy [3]–[13], [65]. It should be noted that the movements underlying our analysis of dynamical changes in image parameters represent approximations to real movements in natural environments. Our simulated rotational movements are the result of rotations of our panoramic images. These software rotations represent “real” rotations sufficiently well, especially with respect to their saccade-like dynamics [4]–[5]. This may be different for our simulated translational movements for technical reasons. Our camera systems could not be moved smoothly at 1 m/s (our simulated velocity) under outdoor conditions, but only stepwise. Images were taken at 1-cm distances and at time steps of 2 s along straight trajectories. Thus, the translational image sequences obtained in this way would correspond to the corresponding real motion sequence only if the visual scenery did not change, while taking subsequent images (e.g. no brightness changes due to clouds occluding the sun or movements of leaves, etc.). This, however, is an unlikely assumption. The “environmental noise” may introduce high frequency components to the fluctuations of image parameters that are evoked by translational movement. Our simulated rotational movements were based on single snapshots and, therefore, did not contain any environmental noise. However, since environmental noise does not decrease the fluctuations during simulated translations, our main conclusion that fluctuations of stimulus parameters during translations in cluttered environments with natural depth structure are smaller than those induced by saccade-like rotations is not affected by the way we constructed our motion stimuli.

In general, translational movements are usually found to cause much smaller changes in image parameters than rotations with saccade-like dynamics. Both amplitude and speed of fluctuations in image parameters differ by about one order of magnitude. Since only changes induced by translational movements contain information about the spatial structure of surroundings [14], the saccadic flight and gaze strategy of insects has been concluded on the basis of electrophysiological experiments and model simulations to facilitate spatial vision [2], [66]–[71]. If rotational and translational movements were superimposed, those changes in image parameters containing spatial information would be confounded by much larger fluctuations caused by rotations [69].

Distinct changes in image parameters are elicited during translations only by objects nearby (e.g. Fig. 3). Therefore, such changes could be the basis for the detection of behaviorally relevant objects without any need for further image segmentation [71]. By contrast, changes in image parameters evoked by saccade-like rotations have much higher amplitudes and frequencies than those evoked by translations, in particular, because they are not only caused by objects nearby, but also by distant ones. The temporal structure and extent of these saccadic and intersaccadic changes in image parameters define the temporal constraints under which the visual system has to operate under behavioral conditions in natural environments.

### Consequences of image parameter fluctuations for information processing and visual adaptation

The limited coding ranges of photoreceptors and neurons as well as the noisiness of all neural computations constrain how reliably information about the environment can be represented by the nervous system [72]–[75]. Visual systems are thought to adjust such ranges to the current input conditions (e.g. [76]) to make optimal use of their limited operating range. However, the properties of the neural hardware do not only limit the range of stimulus amplitudes that can be processed and represented. The timescale on which time-varying stimuli can be encoded is also constrained by the time constants of neural mechanisms at all processing stages. Photoreceptors, for instance, require several milliseconds to transform light stimuli into electrical signals (e.g. [73], [77]), and motion detection mechanisms rely on time constants to match their output to the velocities that are to be represented [78]–[79]. A close match has been proposed in various studies between the time constants of the different stages of the insect motion vision pathway and the different retinal velocity ranges that might result from the partly different flight style of different insect species [80]–[83]. Moreover, motion detection systems of insects have been shown to adjust their operating range by adaptive mechanism on a wide range of timescales to the prevailing dynamical conditions (reviews [84]–[85]).

Most previous studies addressing the constraints imposed on the operating ranges of visual neurons, their dynamical properties and adaptation processes are based on systems analyses with stimuli designed by the experimenter. They did not usually take visual stimuli into account that approximated those encountered by the animal under behavioral conditions. Hence, the current functional interpretations of operating ranges and adaptation processes in visual neurons can only be tentative. An exception are photoreceptors and first-order visual neurons of flies, which were investigated with natural stimulus sequences that approximated what the animal might have seen outdoors [27], [86]–[88]. However, even in these studies, the brightness fluctuations resulted from smooth camera movements rather than from the saccadic flight style of insects. Thus, they differed from the high-frequency brightness fluctuations evoked by saccades and the smaller and slower fluctuations characteristic of intersaccadic intervals (see Fig. 3C, D and Fig. 7).

The parameters characterizing neural computations are often assumed to be tailored to the statistics of natural visual input in an information-theoretically optimal manner. This idea implies that changes in input statistics, as they might occur during flight, should be reflected by adaptive adjustments of computation parameters to ensure optimal information transmission at any time [89]. Natural input signals are characterized by a high degree of redundancy, whereas neural computation is inherently noisy. Thus, the optimal encoding strategy requires a balance between removing and retaining redundancy depending on the signal-to-noise ratio of the signals that need to be processed. The optimal balance is captured by the principle of maximizing the mutual information between the relevant stimulus features that are encoded and the corresponding responses at the different levels of the visual system [45], [89]–[91]. In this context, however, it is a frequent conceptual problem to decide what the relevant stimulus features are and what computational task is being solved by a particular neural circuit.

Therefore, it can only be assessed how well the visual computations and their adaptive properties are matched to the natural image statistics and, in particular, the image parameter fluctuations during behavioral sequences in case not only the visual input is known, but also the stimulus features that need to be encoded at the different levels of the visual system. Information about what needs to be encoded is – to some extent – obvious for the peripheral visual system (photoreceptors and first-order neurons). Accordingly, models successfully describing the adaptive processing of naturalistic input sequences with a high dynamic brightness range are available [87], [92]. However, it is still an open question what information is encoded further downstream in the visual system. In particular, although we know a lot about the response properties of directionally selective wide-field neurons at the output level of the visual motion pathway to optic flow encountered during free-flight, their functional role in behavioral control is still controversial [2], [84], [93]–[95]. This is especially true with respect to the significance of the pattern-dependent response modulations that have been found in these neurons despite their large receptive fields [71], [96]–[101]. If these wide-field motion-sensitive cells are thought to represent detectors of the animal's self-motion and to play a role in compensating for deviations from flight course, the fluctuations in image parameters evoked during locomotion will just reflect “noise” that should somehow be attenuated by information processing further downstream. However, if these neurons are conceived as providing information about the environment during translational intersaccadic movements, these pattern- and distance-dependent fluctuations are likely to be of functional significance and should be made explicit by downstream information processing (see [2]). In either case, adaptation mechanisms should be tailored in different ways depending on the task the neurons are thought to solve. This issue and, in particular, the question of how visual information processing at the different stages of the visual system is adapted to the spatiotemporal statistics in natural image sequences cannot yet be resolved and requires further studies.

## Supporting Information

Appendix S1 **Time course of first order statistics of the mean brightness and of the root mean square contrast in differently sized image patches during rotational and translational movements (see Appendix S1 for details).** Moreover, we examined the spatial frequency statistics within the image patches and, thus, calculated two-dimensional power spectra (Fig. 2D–F), after having tapered the image patches with a sinusoidal window to eliminate boundary effects. Power spectra were calculated by taking the squared magnitudes of fast Fourier transformations (FFTs). Taking the mean over all frequencies for each viewing direction, we obtained the orientation-dependent power spectra (Fig. 2G) providing some information about the orientations of contours that were most prominent in the image. By taking the mean over all orientations for each frequency, we calculated the frequency-dependent power spectra (Fig. 2H). More details are given in Appendix S1.
(DOCX)
