# Supplementary material for: Temporal Statistics of Natural Image Sequences Generated by Movements with Insect Flight Characteristics
Source: PLoS One. 2014 Oct 23;9(10):e110386. doi: 10.1371/journal.pone.0110386 (PMC4207754; doi:10.1371/journal.pone.0110386)
Supplement: Appendix S1 — Time course of first order statistics of the mean brightness and of the root mean square contrast in differently sized image patches during rotational and translational movements (see Appendix S1 for details). Moreover, we examined the spatial frequency statistics within the image patches and, thus, calculated two-dimensional power spectra (Fig. 2D–F), after having tapered the image patches with a sinusoidal window to eliminate boundary effects. Power spectra were calculated by taking the squared magnitudes of fast Fourier transformations (FFTs). Taking the mean over all frequencies for each viewing direction, we obtained the orientation-dependent power spectra (Fig. 2G) providing some information about the orientations of contours that were most prominent in the image. By taking the mean over all orientations for each frequency, we calculated the frequency-dependent power spectra (Fig. 2H). More details are given in Appendix S1. (DOCX) [file pone.0110386.s001.docx]

**Supporting Information**

**Appendix S1**

The formulas applied to obtain the ***longitude and latitude of a pixel in the target projection*** using the inverse Lambert azimuthal equal-area projection are

$$\phi=\sin^{-1} \left( \cos c\cdot\sin\phi_{1}+\frac{y\sin c\cdot\cos\phi_{1}}{\rho} \right)$$

$$\lambda=\lambda_{0}+\arctan\left( \frac{x\cdot\sin c}{\rho\cdot\cos\phi_{1}\cdot\cos c-y\cdot\sin\phi_{1}\cdot\sin c} \right)$$

with $\lambda$ being the longitude, $\phi$ the latitude and *ρ* the distance from the point of tangency

$$\rho=\sqrt{x^{2}+y^{2}}$$

and *c* substituting

$c=2\arcsin\left( \frac{1}{2}\rho\right)$.

$x$ and $y$ are the Cartesian coordinates of the pixel in the target patch and$\phi_{1}$ is the standard parallel – the central latitude – and $\lambda_{0}$ the central longitude of the patch center. Using latitude and longitude of the target pixel, we calculated with the polyval function – obtained by way of the slightly modified version (the modification was adding the method for finding an inverse function (polyval), further explained in the methods section) of the omnicam-calibration toolbox by Davide Scaramuzza for Mathworks MATLAB 2010b [1]-[3] – the Cartesian coordinates corresponding to the spherical coordinates describing the source ring image.

$$r=\mathrm{polyval}(calib,\phi)$$

and then

$$x=(r\cdot\cos\lambda)+x_{center}$$

$$y=(r\cdot\sin\lambda)+y_{center}$$

Image statistics were calculated according to van der Schaaf and van Hateren [4].

The ***mean brightness*** *l_mean_* of an image patch was obtained by taking the mean of all pixel values

$$l_{mean}= \frac{1}{L}\sum_{x,y} l(x,y)$$

with $l(x,y)$ denoting the intensity of a pixel at the spatial coordinates $(x,y)$ and *L* being the number of pixels in the image patch.

We used a sinusoidal window function (*w*) to avoid boundary effects in later frequency analysis. Window parameters were set in this way so that the center value of the weight matrix was 1 and the square patch was enveloped by a complete sine phase in a way that the values decreased to zero towards the edges.

The ***weighted mean brightness*** *μ* is then given by

$$\mu= \frac{\sum_{(x,y)} l\left( x,y \right) w(x,y)}{\sum_{(x,y)} w(x,y)}$$

The root mean square contrast was then calculated by taking the standard deviation of the brightness of all pixels of the image patch divided by the mean brightness.

$$c_{r}=\frac{\sqrt{\frac{1}{L}\sum_{(x,y)} {(l\left( x,y \right)-\frac{1}{L}\sum_{(x,y)} l(x,y))}^{2}}}{\frac{1}{L}\sum_{(x,y)} l(x,y)}$$

The weighted mean was subtracted to obtain the power spectrum of an image patch. The patch was then normalized and windowed using the sinusoidal windowing function described above. The Fourier transform $F(f,\phi)$ was then calculated using a fast Fourier transformation that gave us the two dimensional spatial frequencies in polar coordinates.

The ***power spectrum***$S(u,v)$, with u as cycles per image and v as orientation, was then obtained by taking the square amplitude of the Fourier transform $F(f,\phi)$ and unfolding it from polar to Cartesian coordinates using linear samples in steps of one degree. High frequencies exceeding the Nyquist boundary were discarded. Low frequencies below 1.5 cycles per image were also discarded because they suffered from discretization artifacts.

The ***1/f-exponent*** (α) was finally calculated by a least square fit of a linear function to $\text{log}(S(u,v))$ after having taken the mean over *u* using the MATLAB® curve fitting toolbox. The negative slope of the linear function equals α. The offset of the line is linearly related to the log contrast [4], but was not further analyzed.

The ***power per orientation*** was calculated by taking the mean of $\text{log}(S(u,v))$ over *v*. Detailed Parameters for the lognormal distributions of the r.m.s. contrasts found in our database, with μ as expected value, and σ^2^ as variance, both obtained by doing a lognormal fit to the distribution, giving the estimated lognormal distribution. n is the number of elements. The other values show the significance obtained with the one-sample Kolmogorov-Smirnoff test, with d_max_ being the maximal deviation of the empirical function from the data and the estimated lognormal distribution and d_α=0.05_ the critical value for the null hypothesis: if d_max_ is larger than this value, the hypothesis H_0_ (the distribution is lognormal) can significantly be rejected. Secondly, d_α=0.95_ is the critical value for the alternative hypothesis: if d_max_ is smaller than this value, the alternative hypothesis H_1_ (the distribution is not lognormal) can significantly be rejected and the null hypothesis H_0_ can be *assumed* (but, however, not proven). Finally the p value as result of the test is given in the last column.

1. Rufli M, Scaramuzza D, Siegwart R (2008) Automatic detection of checkerboards on blurred and distorted images. Proceedings of the IEEE/RSJ International Conference on Intelligent Robots and Systems (IROS 2008), Nice, France: 3121-3126.

2. Scaramuzza D, Martinelli A, Siegwart R (2006) A flexible technique for accurate omnidirectional camera calibration and structure from motion. Proceedings of IEEE International Conference of Vision Systems (ICVS'06), New York, USA: 45.

3. Scaramuzza D, Martinelli A, Siegwart R (2006) A toolbox for easy calibrating omnidirectional cameras. Proceedings of the IEEE International Conference on Intelligent Robots and Systems (IROS 2006), Beijing, China: 5695-5701.

4. van der Schaaf A, van Hateren JH (1996) Modelling the power spectra of natural images: Statistics and information. Vision Res 36: 2759-2770.
